# Localized hamstring bioimpedance in marathon runners is related to muscle high-energy enzyme serum levels and predicts race time

**DOI:** 10.3389/fphys.2024.1337119

**Published:** 2024-03-05

**Authors:** Lexa Nescolarde, Javier Yanguas, José A. Hernández-Hermoso

**Affiliations:** ^1^ Department of Electronic Engineering, Universitat Politècnica de Catalunya, Barcelona, Spain; ^2^ FIFA Medical Center of Excellence, Ciutat Esportiva Futbol Club Barcelona, Medical Department, Futbol Club Barcelona, Barcelona, Spain; ^3^ Department of Orthopedic Surgery and Traumatology, Hospital Universitari Germans Trias i Pujol, Badalona, Spain; ^4^ Department of Surgery, Faculty of Medicine, Universitat Autònoma de Barcelona, Barcelona, Spain

**Keywords:** hamstring, localized bioimpedance, marathon, race time, skeletal muscle biomarker, creatinine kinase, lactate dehydrogenase, reactance

## Abstract

**Introduction:** The aim was to analyze the response of serum levels of inflammatory, high-energy muscle biomarkers and hamstring localized bioimpedance (L-BIA) measurements to marathon running and to ascertain whether they correlate with each other or with race time.

**Methods:** Blood samples and hamstrings tetra-polar L-BIA measurements from 14 Caucasian male recreational athletes at the Barcelona Marathon 2019 were collected at base line, immediately after and 48 h post-race. Serum C reactive protein (sCRP), creatinine kinase (sCK) and lactate dehydrogenase (sLDH) were determined using an AU-5800 chemistry analyzer. L-BIA was obtained at 50 kHz with a Quantum V Segmental phase-sensitive bioimpedance analyzer.

**Results:** Median sCRP increased (4-fold) after 48 h post-race. Median sCK and sLDH levels increased immediately post-race (3-fold, 2-fold) and 48h post-race (5-fold, 1-fold). Left, right and combined hamstring reactance (Xc) and phase angle (PhA) increased immediately post-race. Xc combined hamstring pre- and immediately post-race correlated with race-time and with sCK and sLDH median levels pre-race. Xc combined hamstring pre- and immediately post-race > 15.6 Ω and 15.8 Ω, respectively, predicted the race time of 3:00:00 h.

**Conclusion:** L-BIA reactance (Xc) is an objective direct, real time, easy, noninvasive bioelectrical parameter that may predict muscle and marathon athlete performance.

## 1 Introduction

Hamstring injuries are familiar to runners, especially those training for long-distance races (Edouard et al., 2016; Ekstrand et al., 2016). Marathon running may lead to the accumulation of eccentrically induced hamstring muscle damage (Morgan, 1990; Fyfe et al., 2013). Monitoring hamstring muscular status before, during, or after a marathon run may help establish a runner’s physical shape and performance and reduce muscle injuries.

Marathon running induces inflammation and muscle necrosis (Neidhart et al., 2000). Blood-circulating biomarkers used to monitor inflammation and muscle damage offer a method to analyze muscle metabolic response to physical activity (Radzimiński et al., 2020). Inflammatory serum biomarkers such as C-reactive protein (CRP), an acute-phase inflammatory protein, and high-energy enzyme serum biomarkers such as creatine kinase (CK), a high-energy phosphate enzyme, and lactate dehydrogenase (LDH), a key glycolytic enzyme, basal serum levels are higher in runners (Rumley et al., 1985) and reportedly have a dose-dependent behavior (Kim et al., 2009), increasing the serum concentration with increased training loads (Radzimiński et al., 2020).

Bioimpedance analysis (BIA) provides a direct, real-time, uncomplicated, and noninvasive measurement of the bioelectrical properties of the whole or a localized (L-BIA) area of the body that reflects tissue hydration and cell membrane integrity (Lukaski et al., 2019). BIA parameters at 50 kHz as resistance (R) relate to whole body water, and at this frequency, alternating current flows predominantly in the extracellular space and the extracellular cell fluid (ECF), and this is evident by the substantially low phase angles encountered in all humans. Reactance (Xc) relates to cell membrane and intracellular fluid (ICF) and provides an index of cell population density or mass (Talluri, 1998), and the phase angle (PhA) reflects the ratio of ICF and ECF (Nescolarde et al., 2023).

In sports, whole-body and L-BIA measurements have been applied as evaluation methods for body composition analysis and muscle assessment, respectively. Whole-body BIA in sports has been used to analyze nutritional status and soft tissue composition (Micheli et al., 2014), quantitative body composition (Campa et al., 2023), aerobic fitness (Matias et al., 2022), training effectiveness, performance levels (Di Vincenzo et al., 2020; López-Fernández et al., 2020; Marra et al., 2020), and even talent identification (López-Fernández et al., 2020; Marra et al., 2020). Consistently, whole-body BIA increases in R, Xc, and PhA have been reported (Castizo-Olier et al., 2018) after different types of sports and physical exercise intensity.

Muscle L-BIA analysis has been used for the assessment of muscular structure in single-frequency (50 KHz) electrical impedance myography to identify pathological changes in muscle architecture with reported decreases in R, Xc, and PhA values due to muscle fiber atrophy, connective tissue accretion, fat infiltration, and edema (Rutkove et al., 2008; Rutkove, 2009; Lukaski and Moore, 2012; Cebrián-Ponce et al., 2021). In sports, muscle L-BIA has been used in professional soccer players to assess muscle injuries with a reported decrease in Xc and PhA according to the grade of injury and anatomical location (Nescolarde et al., 2015; Nescolarde et al., 2017) and directly related to the time to return to play (Nescolarde et al., 2020b). The question that still needs an answer is whether L-BIA can be used to monitor specific muscle status and predict performance depending on muscle fluid and cell density or mass adaptations after training.

The study aimed to extend knowledge about the response of serum levels of inflammatory, high-energy enzymes and hamstring L-BIA measurements pre- and post-marathon running in healthy non-elite athletes and ascertain whether they correlate between them and with race time performance. We hypothesized that well-trained marathon runners would increase the metabolism of CRP (inflammatory) and CK and LDH (high-energy enzymes) after a marathon run and that hamstring L-BIA parameters would increase with specific muscle metabolic changes. We further hypothesized that hamstring L-BIA parameters would be related to race time performance.

## 2 Materials and methods

We examined the dynamics of a panel of inflammatory and high-energy metabolic biomarkers and the hamstring L-BIA parameters in non-elite athletes completing the Barcelona Marathon 2019. These data were recorded as part of the SUMMIT Project, whose primary goal is to analyze health risks in the population engaged in high-intensity physical activities (Nescolarde et al., 2020a; Hernández-Hermoso et al., 2021). The procedures followed the ethical standards of the responsible committee on human experimentation (institutional and national) and the Helsinki Declaration of 1975, revised in 2000. The local ethics committee approved the study (approval No PI-17-037), and all participants provided written informed consent.

### 2.1 Participants

A total of 35 runners, 29 male and 6 female, out of a total of 70 invited, responded to a call for volunteers. The participants were excluded if the race time was slower than the third pacemaker (3:15 h) in the Barcelona Marathon 2018 or if 1) they had lower limb joint symptoms 6 months prior to the marathon, 2) they had any history of previous lower limb surgery, or 3) they had taken any medication in the previous month or during the 48 h post-marathon. We recommended no intense physical exercise for the 48 h period before and after the marathon. A checklist was used to supervise the medication, food intake, and exercise constrains; all the runners complied with the checklist. The final sample included 14 healthy Caucasian male non-elite runners (36 (5) years; 76.2 (8.0) kg; 1.80 (0.1) m; 23.5 (2.3) kg/m^2^) that run 6 (IQR: 5–8) h/week and had a running history of 7 (IQR: 5-11) years, on average, before the race.

The Barcelona Marathon 2019 (42.195 km on asphalt at sea level) began at 8:30 a.m. at a temperature of 8°C–9°C, with winds of 13–15 km/h and 55% humidity. During the race, the runners maintained adequate hydration levels by eating and drinking at aid stations every 5 km, according to pre-established guidelines, with mineral water, sports drinks, fruits, and nuts. The first liquid intake was programed at 60 min into the race: 400 mL for lighter/slower runners and 800 mL for heavier/faster runners, drinking 100–150 mL every 15–20 min. Commercialized beverages were provided for runners with an average of 480 mg/L for Na^+^, 85 mg/L for K^+^, and 45 mg/L for Mg^2+^. The race time of our runners was 3:19:35 (IQR: 3:49:39-3:09:00). The race time recorded by each participant was the official time from the race organization (Zurich Barcelona Marathon).

### 2.2 Measurements of inflammatory and high-energy enzyme biomarkers

A certified phlebotomist collected blood samples from an antecubital vein immediately after arriving at the laboratory. The area was cleaned with isopropyl alcohol, then the needle was inserted, and blood was drawn into an 8.5 mL, 16 × 100-mm Vacutainer ^®^ BD^®^ tube with SSTTM II advance gel (reference 5T02.367953). Baseline samples were taken 24 h before the race, immediately post-race, and 48 h post-race. Non-fasting blood samples were collected at the same time of the day (from 11:30 to 13 a.m.). Before the pre and 48 h post-race blood sample draws, the subjects were instructed to have breakfast after overnight fasting with sports drinks, fruits, and nuts from 9 to 10 a.m. The blood samples were centrifuged at 3,000 rpm at 4°C for 10 min in a bench-top centrifuge. The supernatant serum was aliquoted and stored in dry ice until the samples were frozen at −80°C in sealed Eppendorf tubes to avoid evaporation until analysis. The laboratory analysis was done in the IGTP laboratories that adhere to the standards of biosecurity and safety procedures. Before freezing the sample, serum CRP, CK, and LDH were determined using an AU-5800 Chemistry Analyzer (Beckman Coulter Inc., CA, United States).

### 2.3 Localized bioimpedance measurements

**FIGURE 1:**
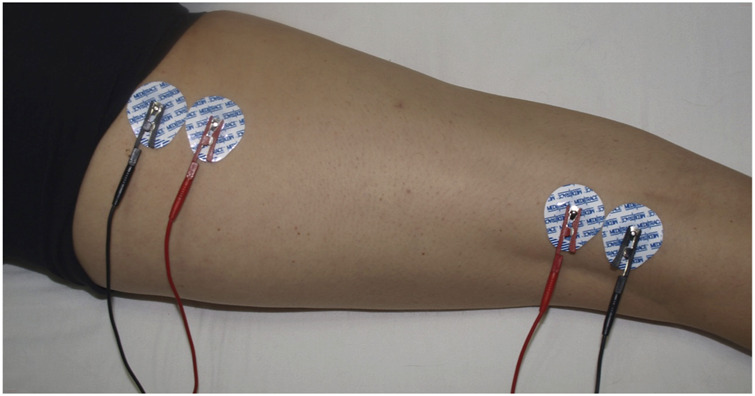
L-BIA measurement in hamstrings. Four Ag/AgCl electrodes, two for the introduction of current (BLUE) and two for detecting voltage (RED), were positioned at the ischiatic tuberosity and immediately proximal to the popliteal line.

Tetra-polar L-BIA measurements were performed 24 h pre-race, immediately post-race, and 48 h post-race, following our protocol for L-BIA measurement on the right and left hamstrings (Nescolarde et al., 2015) to quantify the difference in R, Xc, and PhA values, considering the 24 h pre-race values as a baseline. Four Ag/AgCl electrodes (Covidien Ref. 31050522, Covidien LLC, Mansfield, IL, United States) with an intrinsic value of R and Xc of 10.89 Ω and 0.30 Ω, respectively—two for the injection of current (I) and two for detecting voltage (V)—were positioned. One of the I-electrodes was positioned at the ischial tuberosity, the other was positioned immediately proximal to the popliteal line, and the two V-electrodes were positioned close to the others in line with the hamstring muscle belly. All the anatomical structures were identified by palpation (Figure 1). The skin was cleaned with isopropyl alcohol before measurements were taken. The temperature was 21°C–24.5°C, and the humidity was 36%–38% inside the sanitary facility. The measurements were obtained at 50 kHz using a phase-sensitive bioimpedance analyzer (Quantum V Segmental, RJL Systems Inc., Detroit, MI, United States) operating at a constant, sinusoidal alternating current injection of 245 µARMS. The range of measure for R is 0–1500 Ω and that for Xc is 0–500 Ω, with a 2% maximum tolerance. Measurement errors determined using a parallel RC circuit of a precision resistor and capacitor were <1 Ω for R and <2% for Xc.

### 2.4 Statistical analysis

The normality of the distribution of the variables was checked using the Shapiro–Wilk test, and the homogeneity of variances was checked using Levene’s test. The variables normally distributed are reported as mean (SD), with a 95% confidence interval for the mean (lower and upper bounds). Non-normally distributed data are reported as median, interquartile range (IQR), and minimum and maximum. Friedman test effect size by Kendall’s W value (0.1– < 0.3: small; 0.3– < 0.5: moderate; and ≥ 0.5: large) for non-parametric analysis and partial *Eta* square effect size (0.01– < 0.06: small; 0.06– < 0.14: medium; and ≥0.14: large) for parametric analysis using ANOVA repetitive measurement was calculated (Tomczak and Tomczak, 2014; Serdar et al., 2021).

The sample size was limited to 14 marathon runners due to resource constraints determined by voluntary recruitment and economic resources.

A repeated measures ANOVA test was used to evaluate the effect of the marathon race on biomarkers of inflammation (CRP) and high-energy enzymes (CK and LDH) and on the right, left, and combined right and left hamstring muscle groups’ localized bioimpedance parameters (R, Xc, and PhA) measured 24 h pre-race 1), immediately post-race 2), and 48 h post-race 3), with a multiple comparison test using the Tamhane test. The Friedman test was used for repeated measures of data non-normally distributed with the Wilcoxon test.

In addition, we made Spearman Rho correlations between immediately post-race (2) for 24 h pre-race (1), 48 h post-race (3) concerning 24 h pre-race (1), and 48 h post-race (3) concerning immediately post-race (2) for all the biomarkers and hamstring L-BIA parameters.

The area under the ROC curve (AUC) analysis was used to establish the discriminative Xc combined hamstring L-BIA measurement power capability for predicting a faster race time in the marathon race according to the race time threshold of 03:00:00 h for discriminating between faster runners. A test is considered good, very good, or excellent when the AUC is [0.75, 0.9], [0.9, 0.97], or [0.97, 1], respectively (Fawcett, 2006).

Data were analyzed using statistical software IBM^®^ SPSS^®^ for Windows version 29.0 (IBM Corp., Armonk, HY, USA). The statistical significance was set at *p* < 0.05.

## 3 Results

The 14 study participants completed all data collection time points.

### 3.1 Inflammatory and high-energy enzyme biomarkers

The median sCRP levels slightly decreased (*p* = 0.025) immediately post-race and increased (*p* = 0.001) to four-fold 48 h post-race compared to pre-race.

**FIGURE 2:**
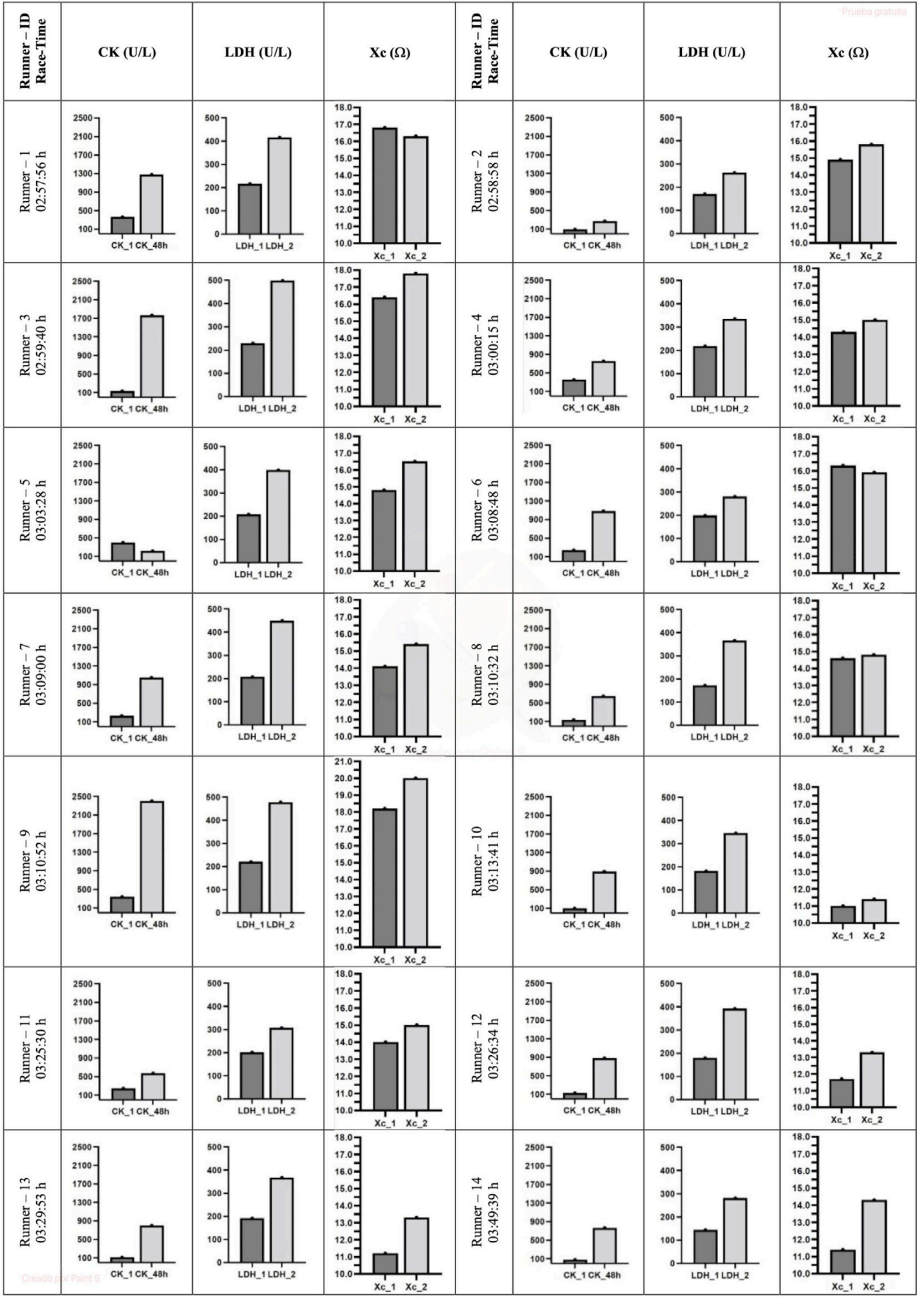
Individual evolution of sCK and sLDH biomarkers and Xc L-BIA. CK, creatine kinase; LDH, lactate dehydrogenase; Xc, reactance. 1, 24 h pre marathon race; 2, immediately post marathon race; 48 h, 48 h post marathon race.

Pre-race median sCK and sLDH levels increased (*p* = 0.001) immediately post-race by up to three-fold and two-fold increases, respectively. The level of sCK remained elevated (*p* = 0.002) 48 h post-race compared to immediately post-race and pre-race (a five-fold increase). In contrast, sLDH median levels decreased (*p* = 0.001) 48 h post-race concerning immediate post-race but remained elevated (*p* = 0.001) concerning pre-race concentration (one-fold increase) (Table 1). A large effect size was observed in all the variables. All the runners showed the same sCK and sLDH behavior, except runner 5, who showed decreased sCK 48 h post-race compared to pre-race levels (Figure 2).

**TABLE 1:** Inflammation and muscle high-energy enzyme serum biomarkers.

Parameter median (IQR) (min–max)	24 h pre-race (1)	Post-race (2)	48 h post-race (3)	Friedman test *p*-value^a^ W effect size	Z *p*-value (2-1)^b^	Z *p*-value (3-1)^b^	Z *p*-value (3-2)^b^	Fold-change (2-1)	Fold-change (3-1)
**CRP (mg/L)** (0–5.0)^†^	1.7 (2.4) (0.3–5.8)	0.9 (2.3) (0.1–4.3)	6.7 (6.0) (2.5–16.3)	24.148 0.000 0.862	−2.237 0.025	−3.297 0.001	−3.296 0.001	∼(−)0.5	∼4
**CK (U/L)** (20–215)^†^	181 (229) (79–396)	612 (286) (271–1393)	839 (504) (214–2401)	19.857 0.000 0.709	−3.296 0.001	−3.170 0.002	−2.794 0.005	∼3	∼5
**LDH (U/L)** (135–225)^†^	200 (39) (144–229)	366 (123) (262–498)	262 (71) (233–448)	28.000 0.000 1.000	−3.296 0.001	−3.297 0.001	−3.297 0.001	∼2	∼1

Data on 14 non-elite 2019 Barcelona Marathon runners. (1), 24 h pre-marathon race. (2), immediately post-marathon race. (3), 48 h post-marathon race.

^a^ Friedman test.

^b^ Wilcoxon test; IQR, interquartile range; min, minimal value; max maximal value; *p*, statistical significance; W, Friedman test effect size (Kendall’s W value).

CRP, C-reactive protein; CK, creatine kinase; LDH, lactate dehydrogenase.

^†^Reference values.

### 3.2 L-BIA hamstring measurements

The left, right, and combined hamstring R did not change immediately post-race or 48 h post-race, concerning pre-race values. In contrast, the measured left, right, and combined Xc (*p* = 0.012, *p* = 0.009, and *p* = 0.003, respectively) and PhA (*p* = 0.010, *p* = 0.011, and *p* = 0.004, respectively) increased immediately post-race with respect to pre-race levels and returned to pre-race levels 48 h post-race (Table 2). A large effect size was observed in all the variables except in R right hamstring, which was small. All runners showed the same Xc behavior, except runners 1 and 6, who showed a decrease in Xc immediately post-race (Figure 2).

**TABLE 2:** Hamstring muscle localized bioimpedance measurements.

Parameter mean (SD) 95% CI for mean lower-bound upper-bound	24 h pre-race (1)	Post-race (2)	48 h post-race (3)	F *p*-value^a^ partial η^2^	SE *p*-value (2-1)^b^	SE *p*-value (3-1)^b^	SE *p*-value (3-2)^b^
**Left hamstring**							
**R-left (Ω)**	50.41 (5.30) 47.35 53.47	50.44 (6.06) 46.95 53.94	52.14 (7.05) 48.07 56.20	1.673 0.229 0.218	0.986 1.000	0.912 0.241	1.389 0.733
**Xc-left (Ω)**	13.67 (2.11) 12.45 14.89	14.72 (1.72) 13.73 15.72	13.64 (2.41) 12.24 15.03	7.482 0.008 0.555	0.301 0.012	0.438 1.000	0.394 0.049
**PA-left (°)**	15.31 (2.91) 13.64 16.99	16.44 (2.52) 14.98 17.89	14.85 (3.04) 13.10 16.60	12.102 0.001 0.669	0.315 0.010	0.460 0.995	0.383 0.003
**Right hamstring**							
**R-right (Ω)**	52.93 (7.18) 48.78 57.07	52.26 (5.91) 48.84 55.67	52.85 (5.76) 49.52 56.18	0.263 0.773 0.042	1.068 1.000	1.236 1.000	1.013 1.000
**Xc-right (Ω)**	14.82 (2.47) 13.39 16.25	15.90 (2.56) 14.42 17.38	14.21 (2.77) 12.61 15.82	8.516 0.005 0.587	0.296 0.009	0.436 0.562	0.469 0.010
**PA-right (°)**	15.91 (3.60) 13.83 17.99	17.05 (3.03) 15.30 18.80	15.19 (3.18) 13.35 17.02	13.867 0.001 0.698	0.321 0.011	0.511 0.532	0.421 0.002
**Left and right hamstrings**							
**R (Ω)**	51.67 (6.10) 48.15 55.19	51.35 (5.69) 48.06 54.64	52.49 (6.27) 48.87 56.11	0.528 0.603 0.081	0.899 1.000	0.926 1.000	1.120 0.979
**Xc (Ω)**	14.25 (2.25) 12.95 15.55	15.31 (2.09) 14.10 16.52	13.93 (2.55) 12.45 15.40	11.015 0.002 0.647	0.254 0.003	0.424 1.000	0.404 0.013
**PA (°)**	15.62 (3.24) 13.74 17.49	16.75 (2.74) 15.16 18.33	15.02 (3.07) 13.25 16.79	16.631 0.000 0.735	0.292 0.004	0.470 0.684	0.381 0.002

Data on 14 non-elite 2019 Barcelona Marathon runners. (1), 24 h pre-marathon race. (2), Immediately post-marathon race. (3), 48 h post-marathon race.

SD, standard deviation; CI, coefficient interval; F, the ratio of the variance between the groups to the variance within the groups; P, statistical significance; partial η^2^ **,** partial *Eta* square.

^a^ Repeated measures ANOVA test.

^b^ Bonferroni test.

R, resistance; Xc, reactance; PhA, phase angle.

### 3.3 Correlation between inflammatory and high-energy biomarkers, hamstring L-BIA, and race time

Xc combined hamstring L-BIA pre-race and immediately post-race was correlated with the race time (*r* = −0.732 and *p* = 0.003) (*r* = −0.682 and *p* = 0.007), sCK (*r* = 0.547 and *p* = 0.043) (*r* = 0.568 and *p* = 0.034), and sLDH (*r* = 0.598 and *p* = 0.024) (*r* = 0.689 and *p* = 0.006) median levels pre-race, respectively (Table 3).

**TABLE 3:** Correlations between Xc-LBIA combined hamstrings and race time, CK, and LDH.

	Xc-1	Xc-2
**Race time**	−0.732** 0.003	−0.682** 0.007
**CK-1**	0.547* 0.043	0.598* 0.024
**LDH-1**	0.568* 0.034	0.689** 0.006

Spearman’s Rho correlation coefficient and significance (2-tailed) on 14 non-elite 2019 Barcelona Marathon runners.

CK, creatine kinase; LDH, lactate dehydrogenase; Xc, reactance.

(1),24 h pre-marathon race. (2), immediately post-marathon race. *p < 0.05; **p < 0.01; ***p < 0.001.

### 3.4 Discriminative capacity of combined Xc hamstring L-BIA for predicting race time

**FIGURE 3:**
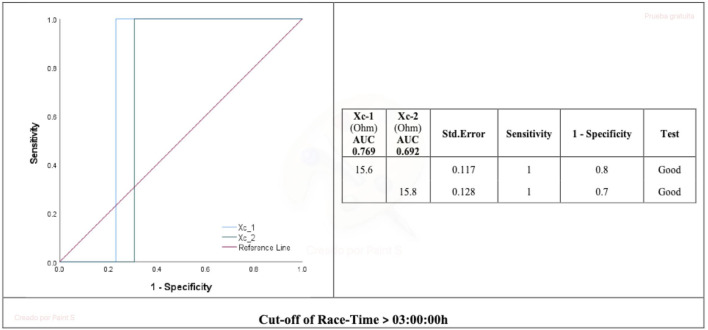
AUC for Xc hamstring localized bioimpedance and race time. Xc_1, reactance 24 h pre marathon race; Xc_2, reactance immediately post marathon race. AUC, area under the curve.

The Xc combined hamstring L-BIA pre-race and immediately post-race >15.6 Ω and 15.8 Ω, respectively, were good cut-off values to predict the race time of 3:00:00 h (AUC = 0.769 and 0.692, respectively) (Figure 3).

## 4 Discussion

Long-distance running exercise imposes high stress on the lower limb musculoskeletal system (Neidhart et al., 2000). Hamstring injuries are familiar to runners, especially those training for long-distance races (Edouard et al., 2016; Ekstrand et al., 2016). There appears to be no specific biomarker that monitors the general or muscular status before running that predicts race performance or predicts muscular cramps or injuries after running.

Our data confirm the results of previous studies (Rumley et al., 1985; Kim et al., 2009; Hernández-Hermoso et al., 2021) that show the variation in inflammation (CRP) and high-energy (sCK and sLDH) biomarkers as a response to intense exercise. We observed an increase in the Xc and PhA values of the left, right, and combined L-BIA hamstring muscles immediately after a marathon run that returned to baseline levels after 48 h. Furthermore, the pre-race and immediately post-race Xc of combined L-BIA hamstring muscle compared to pre-race sCK and sLDH median levels and pre-race and immediately post-race combined L-BIA hamstring muscle Xc of 15.6 Ω and 15.8 Ω predicted a faster race time.

### 4.1 Inflammatory and high-energy enzyme biomarkers

Strenuous exercise can result in muscle damage (Clarkson, 2007; Carmona et al., 2015), which is particularly exacerbated if eccentric contractions are performed (Morgan, 1990; Proske and Allen, 2005; Fyfe et al., 2013). Hamstring eccentric forces are high during the sprinting cycle, especially at the terminal swing phase (Schache et al., 2010). Repetitive eccentric contractions associated with running may lead to the accumulation of eccentrically induced muscle damage (Morgan, 1990; Fyfe et al., 2013). The magnitude of the muscle damage is primarily reduced when the same or similar eccentric exercise is repeated within several weeks (McHugh, 2003).

Running a marathon induces hamstring inflammatory edema after days 1 and 3, appearing as T2 increases on MRI in the hamstring muscles and greater in the distal and middle regions than in the proximal region; the T2 signal reportedly returns to normal by day 8 (Higashihara et al., 2020). The hamstring muscle hardness may increase after a marathon and apparently return to baseline 8 days after the race (Inami et al., 2018). Eccentric exercise leads to structural signs of muscle damage (Proske and Morgan, 2001), as reflected by muscle electron microscope examinations after eccentric exercise in rats. These include (Morgan and Allen, 1999) 1) sarcomeres out of register with one another, 2) Z-line streaming, 3) regions of overextended sarcomeres or half-sarcomeres, 4) regional disorganization of myofilaments t-tubule damage that may involve the elastic filament titin, which anchors thick filaments to Z discs, or the structural protein desmin, which links adjacent Z discs. After a week of eccentric exercise in rats, muscular fascicle length increases (Timmins et al., 2016), and the number of sarcomeres increases by up to 11% (Lynn et al., 1998).

An up to 10-fold increase has been observed in sCRP after day 2 of a marathon run (Hernández-Hermoso et al., 2021). A 3.4-fold increase in plasma hs-CRP has also been observed after day 1 in marathon runners (Kim et al., 2009); the increase returns to the pre-race level on day 4, with a dose-dependent behavior reflected by a higher increase in ultra-marathon runners (200 km). CRP is an acute-phase protein produced in the liver (Rhodes et al., 2011) that can function as an opsonin by coating particles and dying cells to facilitate their uptake by phagocytic cells (Gershov et al., 2000; Newling et al., 2019). It is, therefore, reasonable to presume that runners with higher cell damage caused during the intense exercise (Neidhart et al., 2000) will have higher levels of sCRP (Hernández-Hermoso et al., 2021).

Creatine kinase reaction is involved in the high-energy phosphate metabolism of cells in tissues with high-energy demands, such as muscle, heart, brain, and kidney. All CK isoenzymes catalyze the reversible transfer of the guanidino group of creatine (Cr) to yield adenosine diphosphate (ADP) and phosphoryl creatine (PCr) (Wyss and Kaddurah-Daouk, 2000). Our data suggest that CK temporarily increases 3-fold in plasma immediately after a marathon run, with a further increase after day 2 (5-fold) (Hernández-Hermoso et al., 2021). Kim et al., 2009 observed a temporary 3-fold increase during and after marathon running, with a further increase after day 1 that returns to pre-race levels after day 6. The decrease in sCK serum levels observed in runner number 5 after 48 h post-marathon may be due to distinct endurance training (Wyss and Kaddurah-Daouk, 2000).

Lactate dehydrogenase is a key glycolytic enzyme released from many tissues that is believed to be a major enzyme responsible for pyruvate-to-lactate conversion. Our data show a transient increase in serum up to 2-fold immediately after a marathon run (Hernández-Hermoso et al., 2021), which may be due to increasing energy demands and muscle cell membrane permeability, which occur as cells become energy depleted, or due to muscle cell damage, which occurs after prolonged exercise (Rumley et al., 1985). The increase in total serum LDH activity that occurs after a marathon run is reportedly modified by training (Rumley et al., 1985).

### 4.2 L-BIA hamstring measurements

Whole-body (Campa et al., 2020; Marini et al., 2020) and segmental (Marra et al., 2020) bioelectric properties of the normal population are different from athletes and different between athletes who practice different sports (Campa et al., 2020; Di Vincenzo et al., 2020; Marra et al., 2020) or those who have an early age maturing phase with higher fat-free mass (Koury et al., 2019). They also vary for the individual athlete throughout the competitive season (Campa et al., 2020; Reis et al., 2020). The changes in bioelectric data reflect body composition. Physical exercise, training load, and nutritional strategies all result in fluid shifts in the body compartments. Independent of their research design, current short-term BIA studies indicate an increase in R and Xc and a significant PhA vector migration after sufficient prolonged and intense physical exercise (Castizo-Olier et al., 2018). R is directly affected by intra- and extra-cellular ion solutions and is related to the hydration status of soft tissues, and PhA is associated with the rate of ICF and ECF(Lukaski et al., 2019; Campa et al., 2020; Marini et al., 2020). Xc relates to the capacitive component of tissue interfaces and cell membranes and, thus, is sensitive to muscle structure integrity (Talluri, 1998). Meleleo et al. (2017) attributed the increase in Xc and PhA observed in swimmers and gymnasts after 6 months of training to improving muscular trophism with higher levels of intracellular proteins and glycogen.

In contrast to whole-body or segmental BIA and serum biomarkers, L-BIA permits the analysis of a specific muscle injury in professional soccer players (Nescolarde et al., 2015; Nescolarde et al., 2017; Nescolarde et al., 2020b), being able to detect the type and degree of muscle connective tissue disruption through the decrease in Xc and the fluid distribution changes through the decrease in PhA. The Xc L-BIA percentage of change is also useful in predicting the time to return to play after the muscle injury (Nescolarde et al., 2020b). The hamstring L-BIA increase in Xc and PhA observed immediately after the marathon run may be related to the structural changes described in muscle fiber sarcomeres after repetitive eccentric exercise (Proske and Morgan, 2001) or a state of maintained muscle contraction where sarcomeres are shorter and the myofilaments proteins are more packed, as reflected by the hardening of the hamstrings after a marathon (Inami et al., 2018). These immediate post-marathon increases in Xc are observed in all runners, except in two. The Xc percentage decrease observed after the race in runners 1 and 6 (−3%) was lower than that reported as a grade I muscle injury (−17.5%) (Nescolarde et al., 2015). Such a decrease may reflect functional muscle damage observed with standard magnetic resonance imaging and without a myo-connective muscle injury (Pollock et al., 2014).

### 4.3 Correlation between inflammatory and high-energy biomarkers, hamstring L-BIA, and race time

The observed correlation of sCK and sLDH pre-race with Xc pre-race and immediately post-race may be related to the increase in muscle high-energy enzymes that are needed for muscle contraction. The hamstring muscle of a well-trained athlete may have an increase in the muscle fiber length (Timmins et al., 2016) and in the number of sarcomeres (Lynn et al., 1998; Proske and Morgan, 2001), as has been observed in rats after repetitive eccentric exercise. The increase in muscle fiber cell lengths and in the number of sarcomeres may increase the intra-cytoplasmic proteins and may change the ICF. These molecular structural changes may cause an increase in Xc and PhA. This last observation is consistent with the increase in Xc and PhA observed in swimmers and gymnasts after 6 months of training, which was also attributed to the higher levels of intracellular proteins and glycogen (Meleleo et al., 2017).

The higher Xc cut-off pre- and immediately post-race observed after a faster marathon run may be because more trained runners may have a hamstring muscle with significantly larger muscular fibers and a higher number of sarcomeres, thus having a higher concentration of intracellular proteins than non-trained ones. It makes sense that an increase in hamstring fiber length, sarcomere number, and cytoplasmic proteins may be related to the serum levels of high-energy enzymes (sCK and SLDH) needed to obtain the necessary energy for muscle contraction and muscle capacitance, as determined by Xc L-BIA. We speculate that the Xc incremented values are due to a reduced interstitial fluid coupled with negligible muscle cell hypertrophy and sarcomere coupling (pairing).

We observed several limitations. First, we had a limited sample size with no control group of less-trained marathon runners. Still, even with this small sample size, we found significant differences and strong correlations. Second, although we documented the diet, hydration, and exercise constraints of the participants in the 48 h period before and after the marathon, we did not have the runners under strict supervision in a sports center, and that can be a limitation for the strict follow-up of diet, hydration, and exercise constraints by the runners.

### 4.4 Conclusion

We confirmed previous studies that observed a transient increase in serum levels of high-energy enzymes (CK and LDH) immediately after a marathon run and an increase in inflammatory proteins (CRP) 48 h post-race. The pre-race serum levels of high-energy enzymes (sCK and sLDH) are related to hamstring Xc pre- and immediately post-race. L-BIA hamstring Xc pre- and immediately post-race greater than 15.6 Ω and 15.8 Ω may predict a fast (<3:00:00 h) race time. High-energy enzyme serum levels and Xc may be related to hamstring muscle cell mass, sarcomeres number, and cytoplasmic myofilament protein concentration. The data suggest that direct, real-time, noninvasive bioelectrical parameters may predict muscle and marathon athlete’s performance.

## 5 Scope statement

The practice of marathon running and sports in general has increased in the last 15 years. Monitoring muscular status before, during, or after a marathon run may help establish a runner’s physical shape and performance and reduce muscle injuries. We observed that high-energy enzymes (CK and LDH) serum levels and reactance (Xc) determined by localized bioimpedance are related and that a reactance greater than 15.6 Ω and 15.8 Ω may predict a fast (<3:00:00 h) race time. We speculate that high-energy enzymes and reactance may be related to muscle cell mass, sarcomeres number, and cytoplasmic myofilament protein concentration. Localized bioimpedance reactance is a direct, real-time, noninvasive bioelectrical parameter that may predict muscle and marathon athlete’s performance.

## Data Availability

The raw data supporting the conclusion of this article will be made available by the authors, without undue reservation.

## Abbreviations

sCRP, serum C-reactive protein; sCK, serum creatine kinase; sLDH, serum lacto dehydrogenase; BIA, bioimpedance analysis; L-BIA, localized bioimpedance analysis; ECF, extracellular fluid; ICF, intracellular fluid; R, resistance; Xc, reactance; PhA, phase angle; AUC, area under the ROC curve.
